# A common tRNA modification at an unusual location: the discovery of wyosine biosynthesis in mitochondria

**DOI:** 10.1093/nar/gkv286

**Published:** 2015-04-06

**Authors:** Paul J. Sample, Luděk Kořený, Zdeněk Paris, Kirk W. Gaston, Mary Anne T. Rubio, Ian M.C. Fleming, Scott Hinger, Eva Horáková, Patrick A. Limbach, Julius Lukeš, Juan D. Alfonzo

**Affiliations:** 1 Department of Microbiology and The Center for RNA Biology, The Ohio State University, Columbus, OH 43210, USA; 2 Institute of Parasitology, Biology Centre and Faculty of Sciences, University of South Bohemia, 37005 České Budějovice (Budweis), Czech Republic; 3 Rieveschl Laboratories for Mass Spectrometry, Department of Chemistry, University of Cincinnati, Cincinnati, OH 45221, USA; 4 Canadian Institute For Advanced Research, Toronto, ON M5G 1Z8, Canada; 5 Ohio State Biochemistry Program, The Ohio State University, Columbus, Ohio 43210, USA

## Abstract

Establishment of the early genetic code likely required strategies to ensure translational accuracy and inevitably involved tRNA post-transcriptional modifications. One such modification, wybutosine/wyosine is crucial for translational fidelity in Archaea and Eukarya; yet it does not occur in Bacteria and has never been described in mitochondria. Here, we present genetic, molecular and mass spectromery data demonstrating the first example of wyosine in mitochondria, a situation thus far unique to kinetoplastids. We also show that these modifications are important for mitochondrial function, underscoring their biological significance. This work focuses on TyW1, the enzyme required for the most critical step of wyosine biosynthesis. Based on molecular phylogeny, we suggest that the kinetoplastids pathways evolved via gene duplication and acquisition of an FMN-binding domain now prevalent in TyW1 of most eukaryotes. These findings are discussed in the context of the extensive U-insertion RNA editing in trypanosome mitochondria, which may have provided selective pressure for maintenance of mitochondrial wyosine in this lineage.

## INTRODUCTION

A defining feature of all tRNAs is the presence of numerous post-transcriptional chemical modifications (1,2). Because some modifications are common to all tRNAs in all domains of life, it has been suggested that such ‘primordial’ nucleosides were essential in ensuring reading-frame maintenance early in the evolution of translational systems (3–7). Although many positions in a tRNA can affect translational accuracy, position 37 of the anticodon loop plays an important, if not a critical, role in reading-frame maintenance (6,8,9). This universally modified position may harbor simple base methylations (such as 1-methylguanosine, m^1^G), which in the context of a specific tRNA anticodon and depending on the inherent stability of anticodon-codon interactions, may be sufficient to prevent translational errors. Other tRNAs may require more complex chemical groups such as N6-isopentenyladenosine (i^6^A) and N6-threonyladenosine (t^6^A) (10–12).

**Figure 1.**
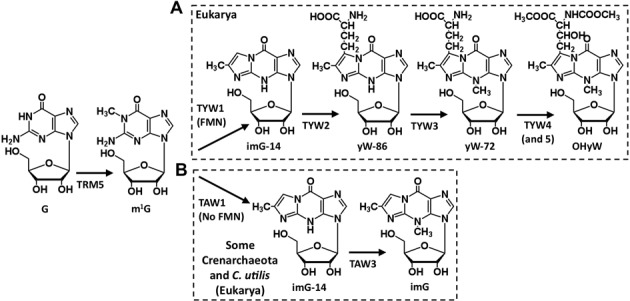
Biosynthetic pathways for wyosine/wybutosine and derivatives. (**A**) The eukaryotic hydroxywybutosine (OHyW). (**B**) the proposed biosynthetic pathway of archaeal (specifically Crenarchaeota) wyosine (imG), also found in the cytoplasm of one eukaryote (*Candida utilis*) (13). TAW1 and TAW3 are the archaeal homologs of eukaryotic TYW1 and TYW3. Eukaryotic TYW4 and TYW5 have never been found in Archaea. Most Euryarchaeota contain a TYW2 homolog that forms yW-86 derivatives. All steps other than hydroxylation of yW-72 via TYW5 require SAM (9).

One of the most chemically intricate modifications that occur at position 37 of the anticodon loop involves the nucleoside wyosine (imG) and its derivatives, including wybutosine (yW) and hydroxywybutosine (OHyW) (5). Wyosine derivatives are exclusively found in the single tRNA^Phe^_GAA_ of Archaea and Eukarya, but despite its omnipresence, the pathway varies greatly between these organisms. The eukaryotic pathway involves at least four sequential reactions that use 1-methylguanosine (m^1^G_37_), as a precursor (13). The m^1^G_37_ modification is itself the product of a phylogenetically widespread methylation found in all domains of life (14–17). Formation of m^1^G_37_ is followed by the incorporation of the C-1 and C-2 carbons of pyruvate by the radical-SAM enzyme TYW1 to form an additional ring on the methylated purine (18,19). The resulting product constitutes the minimal core of the modification, with 4-demethylwyosine (imG-14) as its simplest form (19). What follows this core tricyclic component is a series of reactions that vary among different organisms. For example, in most eukaryotes the end product of the reaction is the nucleoside wybutosine (yW) (not shown in Figure 1A), the product of 3 additional enzymes in yeast (TYW2–TYW4) (13). In some organisms, including humans, yW can be further modified by a fifth enzyme, TYW5, to hydroxywybutosine (OHyW) (20) (Figure 1A).

In Archaea, the situation varies even more, with most species having at least one type of wyosine derivative ranging from 4-demethylwyosine (imG-14, the product of TAW1) to isowyosine (imG2), wyosine (imG), methylwyosine (mimG), 7-carboxypropyl-demethylwyosine (yW-86) and/or 7-aminocarboxypropylwyosine (yW-72) (21). This diversity is created by TAW1, TAW2 and TAW3, which can act in a strictly sequential pathway or in a combinatorial manner. Among these, most striking is the presence of a pathway shared by some Crenarchaeota (Figure 1B), which lacks TAW2 and involves only TAW1 and TAW3, yielding imG as the end product (21).

Wyosine and derivatives are thus far absent in Bacteria and instead bacterial tRNA^Phe^ has an encoded A_37_, which then can be modified to i^6^A (or ms^2^i^6^A), given their shared ancestry, the same is expected of mitochondria (6,22,23). However, in protists such as *T. brucei*, there is a complete lack of tRNA genes in the mitochondrial genome (24). These organisms are therefore forced to import all of their tRNAs from the cytosol for organellar translation; their strategy for reading frame maintenance during translation then becomes of particular interest.

In the present study, we report two distinct pathways for wyosine biosynthesis and its derivatives in trypanosomatids: one cytosolic and the other mitochondrial. The coexistence of these two pathways is made possible by the trypanosomatid-specific presence of two TYW1 enzymes yielding different wyosine derivatives as end products: wybutosine/hydroxywybutosine, found in the cytosol and mitochondrion, and wyosine, strictly confined to the mitochondrion. This represents the first example of an organellar wyosine pathway with implications for how the pathway evolved in eukaryotes and suggesting a new strategy for translational fidelity in mitochondrial systems.

## MATERIALS AND METHODS

### RNA interference

Portions of the coding sequence of the TYW1L and TYWS genes from *Trypanosoma brucei* were cloned into the tetracycline-inducible RNAi vector p2T7-177. These plasmids were then linearized by NotI digestion (for genomic integration) and transfected into procyclic *T. brucei* 29-13 cells and clonal lines were obtained by limiting dilution, as described elsewhere (25). RNAi was induced after the addition of 1 mg/ml of tetracycline to SDM-79 growth medium. Cell counts were taken every 24 h using the Beckman Z2 Coulter counter over the course of 10 days post-induction. In these growth curves, the induced sample was compared to a non-induced control grown in the absence of tetracycline and a similar growth curve performed using wild type cells. Targeted gene down-regulation was confirmed by RT-PCR, as described below.

### RT-PCR and qPCR

Total *T. brucei* RNA was isolated using standard protocols RNA (5 μg) was used for all RT-PCR reactions following the manufacturer instructions (Invitrogen). Reverse primers specific for TYW1L (5′-TGATGACATCATCATTAGAGGAGC-3′) and TYW1S (5′-GTCGTGTGGGTTATCGACTTGC-3′) were used to generate cDNA. The resulting cDNAs were used in PCR amplification reactions using the same reverse primers, a forward primer specific for TYW1L (5′-CGTTGGCTGTTGGGTGTTAACG-3′), and a forward primer specific for the 5′ UTR of TYW1S (5′-TCGCCCGATGCTTTCGGAGC-3′). Products were then analyzed by agarose gel electrophoresis. For each RNAi experiment the complete set of 4 primers (for TYW1L and TYW1S, respectively) were used to ensure that the RNAi down-regulation of one of the genes did not affect the other, ruling out off-target effects. A similar reaction incubated in the absence of reverse transcriptase served as a negative control (RT-) and as a control for DNA contamination. Quantitative real-time PCR was performed with total RNA isolated from each cell line as described previously (26). The QuantiTect Reverse Transcription Kit (QIAGEN) and Oligo (dT)_20_ (invitrogen) were used to generate cDNA. Quantitative real-time PCR was performed as described elsewhere (10). The primer pair Tyw1S-qPCR-FW (5′GCTCGCTTGTGTGAGACTATAC3′) and Tyw1S-qPCR-RV (5′GTGTGCCAAACACCATCAAC3′), Tyw1L-qPCR-FW (5′CGGCTGACTCTCGTGAATAAA3′) and Tyw1L-qPCR-RV (5′GGTGAGTGTTGATGTGCTACT3′) were used to detect Tyw1S and Tyw1L mRNA respectively. The primer pair of 18SrRNA 18S-qPCR-F (5′GATTTTGGGCAACAGCAGGTC3′) and 18S-qPCR-R (5′CCTACGAGACATTCCTCGTTGC3′) was used as an internal reference.

### Western blot and immunofluorescence

The protein coding regions of TYW1L and TYW1S were separately cloned into the *T. brucei* protein expression vector pLew79-V5. This vector places a V5-epitope tag at the C-terminus of each protein. These constructs were individually transformed into procyclic *T. brucei* 29–13 cells and clonal cell lines were established as described before. Cells were RNAi-induced by the addition of tetracycline for 24 h and either harvested and total cell extracts prepared (as described elsewhere) to use for western blot analysis or intact cells were fixed to a microscope slides and used for immunofluorescence microscopy.

For each construct, one liter of induced cells were harvested at 1 × 10^7^ cells/ml. Fifty milliliters of this culture was sonicated to produce the total cell lysate. The rest of the culture was used to prepare the cytosolic and mitochondrial fractions as described elsewhere (27). Total, cytosolic, and mitochondrial protein fractions (10 μg/lane) were separated on 10% sodium dodecyl sulfate (SDS)-polyacrylamide gel, blotted, and subjected to western blot analysis with the following monoclonal mouse primary antibodies: V5. Secondary anti-mouse IgG antibodies coupled to horseradish peroxidase (GE Healthcare) were use for visualization using the Clarity™ Western ECL system (BioRad) and following the manufacturer's instructions. Rabbit polyclonal antibodies specific for *T*. brucei Isd11 (28) and enolase (kindly provided by P.A.M. Michels) were used as controls for mitochondrial and cytosolic fraction purity, respectively. The same primary antibodies were used for immunofluorescence, while Alexa Fluor 488-conjugated goat anti-mouse antibodies (Life Technologies) were used as the secondary antibody. For mitochondrial detection, the *T. brucei* cells were stained with 200 nM MitoTracker Red dye (Life Technologies) for 20 min at 27°C, washed, and fixed to slides with 4% paraformaldehyde. Mitochondrial and genomic DNA were stained with DAPI shortly before immunofluorescence microscopy.

### LC-MS/MS analysis

Two liters of *T. brucei* culture at 1 × 10^7^ cells/ml were used for each cell compartment-specific nucleoside analysis. Mitochondria were purified following cell breakage by hypotonic lysis, followed by separation of cell components in Renografin density gradients as described previously (37). One hundred milliliters of the cytosolic RNA fraction were extracted with H_2_O-saturated phenol, 10 ml 2 M sodium acetate, and 33 ml of 50:1 (v/v) mixture of chloroform:isoamyl alcohol, vortexed, chilled on ice for 10 min and the aqueous phase collected following centrifugation at 14 000 x *g f*or 10 min. The RNA in the resulting aqueous phase was precipitated by the addition of an equal volume of isopropanol. After centrifugation at 14 000 x *g*, the cytosolic RNA pellet was resuspended in 1 ml of TE followed by a second extraction with Tris (pH 8.0)–phenol–chloroform mixture followed by ethanol precipitation. Similarly RNA was extracted from the Renografin-gradient purified mitochondria, except that prior to extraction the organelles were treated with 100 U of micrococcal nuclease for 30 min and 37°C to degrade any extramitochondrial RNA. Mitochondrial RNA was then purified using the guanidinium thiocyanate/phenol/chloroform extraction method. Total tRNA from these cytosolic and mitochondrial RNA fractions were purified by 8% polyacrylamide gel electrophoresis followed by passive elution after the excision of the tRNA band from the gel. For nucleoside analysis, 20 μg of tRNA was digested to nucleosides using nuclease P1 (Sigma–Aldrich) and Antarctic phosphatase (New England Biolabs). The nucleosides were separated using a Hitachi D-7000 HPLC with a Hitachi L-7444 diode array detector at a flow rate of 0.3 ml/min at room temperature on a Supelcosil LC-18-S 250 × 2.1 mm column (Sigma–Aldrich). Nucleosides were separated using a combination of two buffers (buffer A: 5 mM ammonium acetate pH 5.3 and buffer B: acetonitrile:water, 40:60 [v/v]). These were used in a step-gradient which is a modified version of a previously described protocol (38): 0 min: 1% B, 5.8 min: 1% B, 9.2 min: 2% B, 10.9 min: 3% B, 12.7 min: 5% B, 32 min: 25% B, 38 min: 50% B, 43.5 min: 75% B, 45 min: 75% B, 50 min: 99% B, 55 min: 99% B, 60 min: 1% B. Column eluent was split into two fractions, with two thirds being analyzed by the UV detector and the remaining one third was injected into an LTQ-XL linear ion trap mass spectrometer (Thermo Scientific). Mass spectra were recorded in the positive ion mode with a capillary temperature of 75°C, spray voltage 4.0 kV and sheath gas, auxiliary gas and sweep gas of 45, 25 and 10 arbitrary units (a.u.), respectively. Data dependent MS/MS of the four most abundant ions were recorded throughout the LC/MS run using a collision energy of 35 a.u.

### Database searches and phylogenetic analysis

Wybutosine has been extensively studied in *Saccharomyces cerevisiae*, but little is known about its biosynthesis in protists. To identify *T. brucei* homologs of the yW enzymes, we performed database searches using *S. cerevisiae* yW protein sequences as BLAST queries for the kinetoplastid genome databases (Tri-Tryp). Four potential reading frames with significant similarities with the *S. cerevisiae* sequences were identified (TbTYW1L, TbTYW1S, TbTYW2, TbTYW3A, TbTYW3B and TbTYW4) (accession number: XP_846333, XP_803801.1, XP_822650.1, XP_829329.1, XP_829699.1 and XP_827514.1, respectively) (Supplementary Figure S1). In addition, a sequence extension in TbTYW4 showed similarity to the TYW5 protein found in some eukaryotes but absent in *S. cerevisiae* (Supplementary Figure S1F). This extension contains a highly conserved Jumonji C (JmjC) domain important for the formation of hydroxywybutosine (OHyW) as the end product of the pathway (20). Fusion of the last two enzymes in the pathway is reminiscent of *Aspergillus oryzae* (Supplementary Figure S1E).

Taw1/Tyw1 protein sequences were aligned using Mafft (29). The N-terminal portion of the eukaryotic Tyw1 proteins that corresponds to the flavodoxin domain was excluded from the alignment. The phylogenetic analysis was performed using Maximum likelihood tree topology and bootstrap branch supports were calculated in RAxML-HPC BlackBox v8 (30) with LG matrix of protein substitution. Bayesian posterior probability values were generated in MrBayes 3.2.2 (31) after 5 000 000 generation with mixed amino acid model and burnin set to 25%.

## RESULTS

### Unique occurrence of paralogous wyosine biosynthesis genes in *T. brucei*

Genome database searches revealed that two of the genes of wyosine biosynthesis (*tyw1* and *tyw3*) were duplicated in the kinetoplastid lineage (which includes the genus *Trypanosoma*). The larger of the two putative TYW1 genes encodes an 835 amino acid (aa) predicted protein we termed TbTYW1L. This protein bears all the conserved features of this family of enzymes including: a flavodoxin-1 domain at the N-terminus needed for FMN binding, two 4Fe–4S iron–sulfur cluster domains, a radical SAM domain, and a recognizable ‘wyosine base formation motif’ (Supplementary Figures S1A, S1B and S2A) (32). The second gene encodes a smaller protein (395 aa) termed here TbTYW1S, which despite containing all the key above-mentioned catalytic domains lacks the flavodoxin-1 domain, a situation analogous to the equivalent archaeal enzyme, TAW1 (Supplementary Figures S1B and S2A) (13,21).

A similar situation occurs with the third enzyme in the pathway TYW3, with two open reading frames of different sequences encoding TbTYW3A and TYW3B (Supplementary Figures S1D and S2A). In yeast TYW3 catalyzes the methylation at N4 of the yW intermediate yW-86, the product of TYW2, to produce yW-72 (13). The archaeal homolog, TAW3, performs the same reaction in species that encode TAW1 and TAW2. In addition, TAW3 may directly methylate imG-14 (the product of TAW1) to form wyosine (imG) as the end product in species that do not encode TAW2 (TYW2 homolog). This last pathway, consisting of a TAW1 (lacking a flavodoxin-1 domain) and TAW3, is found in some Crenarchaeota (Figure 1B), and has so far been described in only one eukaryote, *Candida utilis* (Figure 1B) (33).

The program Target-P predicted TbTYW1L to localize to the cytosol and TbTYW1S to the mitochondrion (Supplementary Figure S2B). The cytosolic localization of TbTYW1L is not unusual given that in all eukaryotes studied so far yW is a nucleo-cytosolic modification. However, the potential localization of TbTYW1S to the single mitochondrion is surprising and would represent the first example of such an enzyme in an organelle.

### Mitochondrial localization of a wyosine modification enzyme

**Figure 2.**
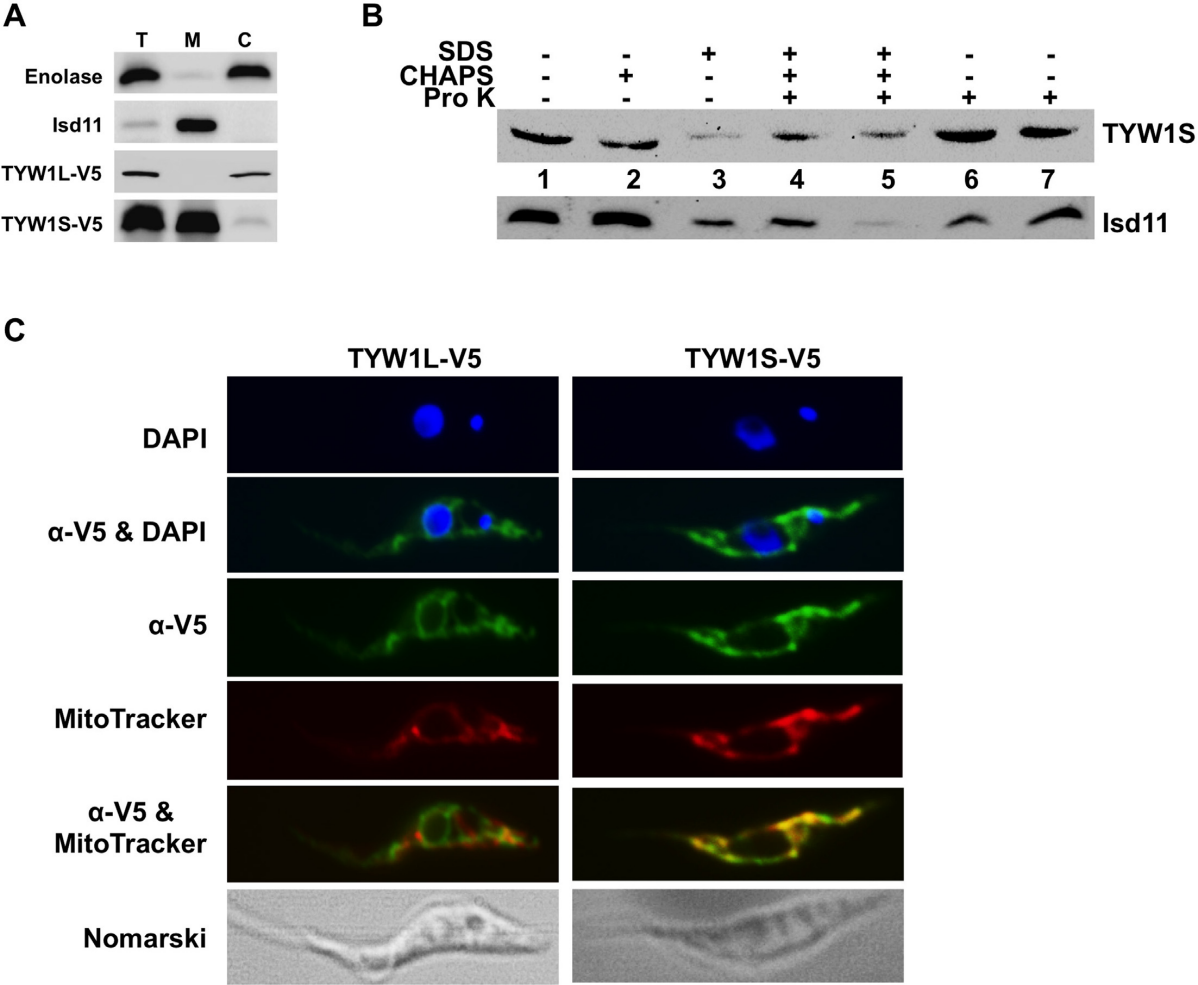
Intracellular localization of TbTYW1L and TbTYW1S. (**A**) Total (T), mitochondrial (M), and cytosolic (C) protein fractions expressing V5-epitope-tagged TbTYW1L and TbTYW1S were analyzed via western blot with V5 epitope-specific antibodies. Similar blots were performed with antibodies specific for *T. brucei* enolase (a cytosolic marker) and Isd11 (a mitochondrial marker). (**B**) Western blots as above, the fractions represent purified mitochondria incubated in the presence or absence of 2 μg (lanes 4 and 6) or 4 μg (lanes 5 and 7) of proteinase K and in the presence or absence of detergents as indicated. Isd11 was used as mitochondrial matrix marker. (**C**) Immunofluorescence-localization experiments with the same V5-antibodies (α-V5) (green fluorescence) and cells expressing V5 epitope-tagged proteins as indicated. DAPI (blue) shows the location of the nucleus (N) and kinetoplast (K). Nomarski refers to a phase-contrast image of the same cells. MitoTracker dye (red) was used as a mitochondrial marker. Yellow fluorescence indicates co-localization of either protein with the MitoTracker dye in merged images (α-V5&MitoTracker).

The predictions in the previous section, if correct, then suggest the existence of wyosine derivatives in mitochondria. To test this idea, we focused on the TYW1L and TYW1S proteins, which represent the first committed step of wyosine biosynthesis. Transgenic cell lines expressing epitope-tagged versions of each protein were generated. Western blot analysis with subcellular fractions (total, cytosolic and mitochondrial) from *T. brucei* expressing C-terminus V5-epitope-tagged TbTYW1L revealed its cytosolic localization (Figure 2A). In these experiments, antibodies against compartment–specific markers (enolase and Isd11 for cytosol and mitochondrion, respectively) were used as localization controls and also as controls for fraction purity (Figure 2A). Similar experiments were performed with V5-epitope-tagged TbTYW1S. Western blot analysis revealed a signal for TbTYW1S in the mitochondrial fraction from transgenic cells (Figure 2A). A faint signal for TbTYW1S is also seen in the cytoplasmic fraction, this is not unexpected given that this protein is made in the cytoplasm and then imported into the mitochondria. In our experience, similar faint signals have been observed with other mitochondria-localized proteins and the presence or absence of the faint signals seems to be protein dependent. Regardless, the TbTYW1S signal observed during Western analysis was not affected by treatment of isolated mitochondria with proteinase K in the absence of detergent, while it disappeared upon addition of detergent (Figure 2B). This pattern of proteinase K resistance was similar to that observed with Isd11, a mitochondria-matrix protein used as a control for mitochondrial localization (Figure 2B). This data further supports the mitochondrial localization of TbTYW1S. These results were further confirmed by immunofluorescence analysis using the same anti-V5 antibody, which confirmed the mitochondrial localization of TbTYW1S-V5, based on its co-localization with the mitochondrion-specific MitoTracker Red dye, leading to yellow fluorescence in the merged images (Figure 2C). Low amounts of yellow fluorescence were also seen with TYW1L, but this was considered negligible given that co-localization is not supported by the Western blot analysis. Taken together, these experiments suggest the existence of two potential pathways for wyosine biosynthesis in *T. brucei*: a typically eukaryotic pathway occurring in the cytosol involving TbTYW1L and a novel mitochondrial pathway potentially involving TbTYW1S.

Additional Western analyses were performed with the remaining putative members of the pathway. These results show that TbTYW3A is cytosolic, while TbTYW3B (the other duplicated gene product) showed mitochondrial localization. In addition, TbTYW4/5 localizes to the cytosol and TbTYW2 localizes to both cytosol and mitochondrion (Supplementary Figures S3 and S2b). Taken together, these experiments support the existence of two potential pathways for wyosine biosynthesis in *T. brucei*: a typically eukaryotic pathway occurring in the cytosol involving TbTYW1L, 2, 3A and 4/5 and a novel mitochondrial pathway potentially involving TbTYW1S, TbTYW2 and TbTYW3B.

### Different wyosine derivatives co-exist in mitochondria

**Figure 3.**
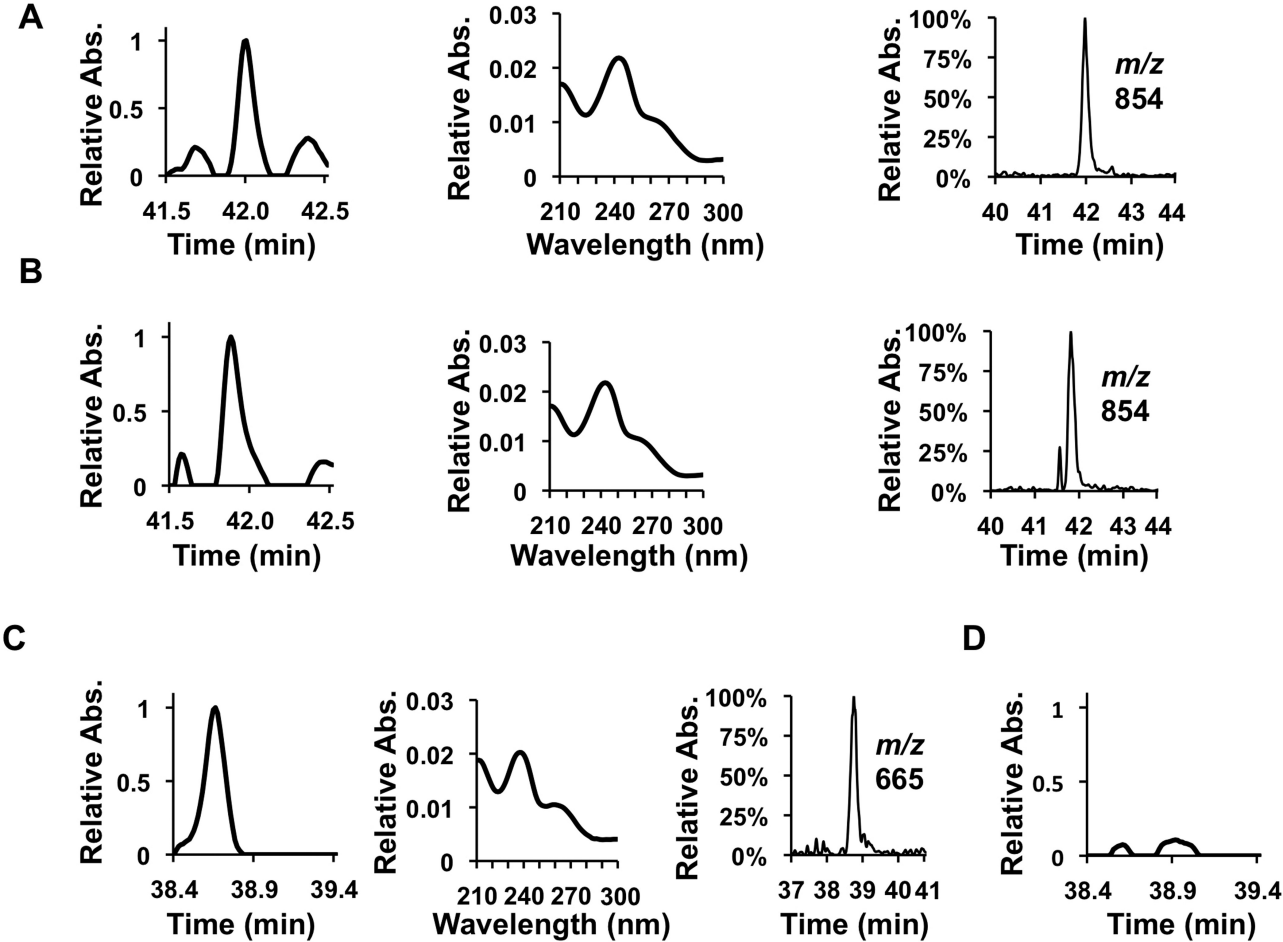
Nucleoside analyses show presence of two wyosine derivatives in *T. brucei* mitochondrion. (**A**) The left panel shows the HPLC UV (254nm) of the total cytosolic nucleosides. The peak eluting at 42 min was further analyzed by UV absorbance showing a spectrum characteristic of hydroxywybutosine/adenosine dinucleoside monophosphate (OHyWpA), the presence of hydroxywybutosine was further confirmed by mass spectrometry *m/z* measurement (right). (**B**) Mitochondrial hydroxywybutosine. Top-left panel, HPLC measurement at 254 nm of peak eluting at 41.9 min, absorbance profile (top-middle), and *m/z* value (top-right) that identify OHyWpA. (**C**) Mitochondrial wyosine. Bottom-left panel, HPLC measurement at 254 nm of peak eluting at 38.7 min, absorbance profile (bottom-middle), and *m/z* value (bottom-right) that confirms presence of wyosine–phosphate–adenosine (imGpA). (**D**) HPLC measurement at 254 nm shows lack of imGpA in the cytosolic tRNA fraction.

**Figure 4.**
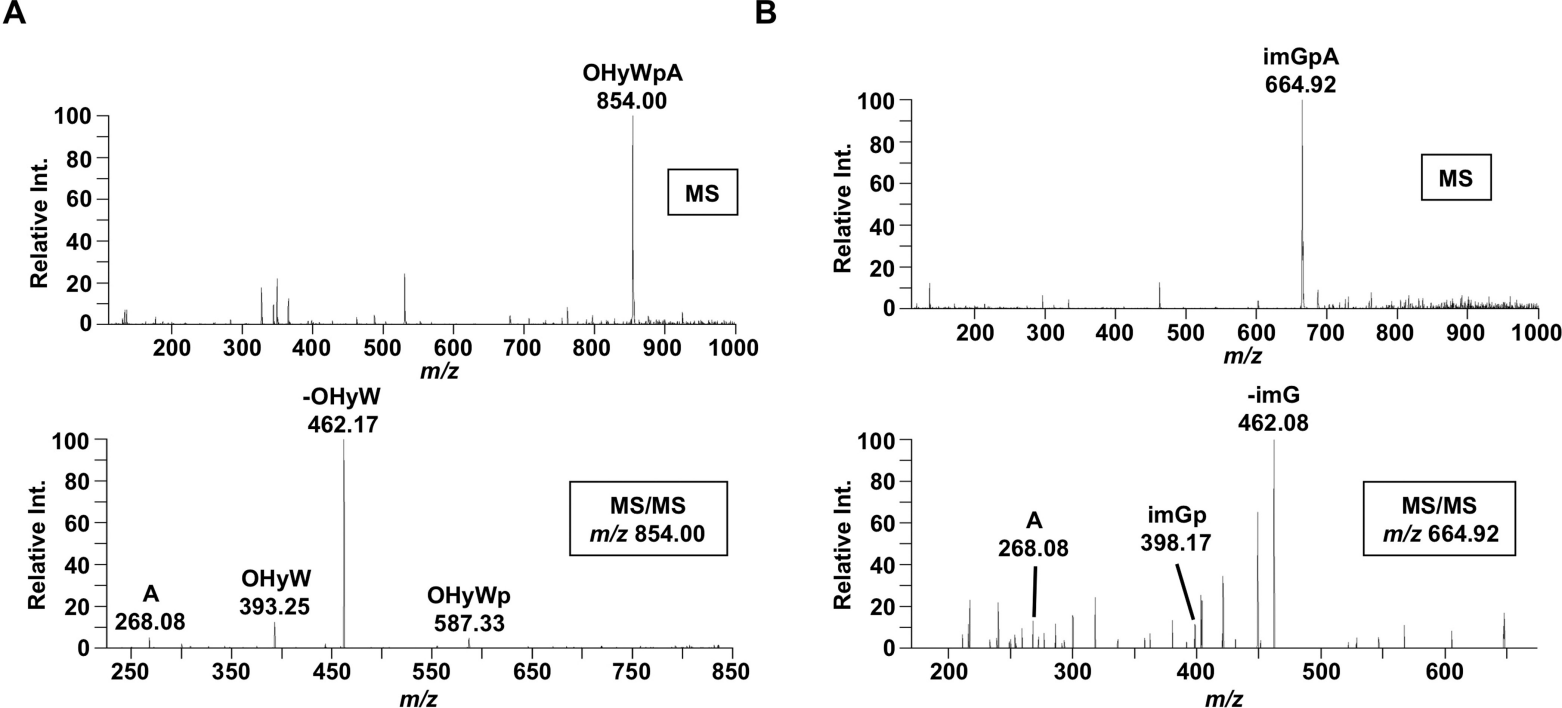
Mass spectral analysis of OHyWpA and imGpA from mitochondrial tRNA. (**A**) To confirm the identity of OHyWpA, the base peak from the mass spectra at 41.9 min with an *m/z* of 854.0 was selected for MS/MS by collision-induced dissociation. The MS/MS produced a predictable fragmentation of this dinucleoside monophosphate including a base loss of OHyW (*m/z* 462.2), adenosine (*m/z* 268.08), OHyW nucleoside (*m/z* 393.2), and OHyWp (*m/z* 587.3). (**B**) The base peak corresponding to imGpA at 38.7 min (*m/z* 664.9) was also analyzed by MS/MS. Although a more complex fragmentation spectrum was obtained, similar peaks found from the fragmentation of OHyWpA were also observed for imGpA, including the loss of the imG base (*m/z* 462.1), adenosine (*m/z* 268.1), and the imG nucleoside (*m/z* 398.2).

To further establish the chemical nature of the wyosine derivatives in *T. brucei*, total cytosolic and mitochondrial tRNA fractions were isolated, digested to nucleosides and analyzed by liquid chromatography tandem mass spectrometry (LC/MS/MS). In the cytosolic tRNA fraction we observed a peak with a mass/charge (*m/z*) ratio of 854, which elutes from the reversed-phase chromatography column with a retention time of 42 min (average: 42.1 min, standard deviation: ±0.15 min.) (Figure 3A). The observed *m/z* value of 854 is consistent with that of a hydroxywybutosine–adenosine dinucleotide with a bridging phosphate (OHyWpA). The appearance of such dinucleotide monophosphate is due to the well-documented resistance of wyosine and derivatives to nuclease P1 digestion used for nucleoside analysis (13). This assignment was further confirmed by its UV-Vis absorbance profile showing a *λ*_max_ at 242 nm and a shouldering peak at 260 nm as has been described elsewhere (18). This dinucleotide monophosphate was also corroborated by collision-induced dissociation tandem mass spectrometry (CID-MS/MS) (Figure 4A). Upon further inspection of the entire UV chromatogram, no other wyosine derivatives were observed in the cytosolic fraction.

The total mitochondrial tRNA fraction showed a peak with an *m/z* value of 854, similar retention time (41.9 min) and comparable UV–Vis absorbance profile as the cytosolic OHyWpA dinucleotide monophosphate (Figure 3B). More importantly, an additional peak was detected with an *m/z* value of 665 (eluting at 38.6 min retention time), which is consistent with the expected values for a wyosine (imG) adenosine dinucleotide monophosphate (imGpA) (Figure 3C). These assignments were confirmed by CID-MS/MS (Figure 4B) and UV–Vis absorbance showing a *λ*_max_ of 237 nm, in line with the published profile for the wyosine dinucleotide (imGpA) (Figure 3C, center panel) (18). However, isowyosine (imG2), an isomer of wyosine, has the same *m/z* value as wyosine but a different HPLC retention time (21). To distinguish between these two possibilities, we purified total tRNA from *C. utilis* and used it as a marker for imG. This yielded nucleosides with identical elution profiles to those of the *T. brucei* mitochondrial fraction (Supplementary Figure S3). This finding was further confirmed by either analyzing the *C. utilis* nucleosides by themselves or by mixing equimolar amounts of the *C. utilis* fraction with the mitochondrial fraction from *T. brucei* (Supplementary Figure S3). The observation of imG in the mitochondrial tRNA fraction suggests that imG may be the product of the sequential reaction of TbTYW1S and the putative mitochondrial version TbTYW3B (Figure 1).

### Lack of TbTYW1L and TbTYW1S leads to corresponding losses of OHyW and imG

**Figure 5.**
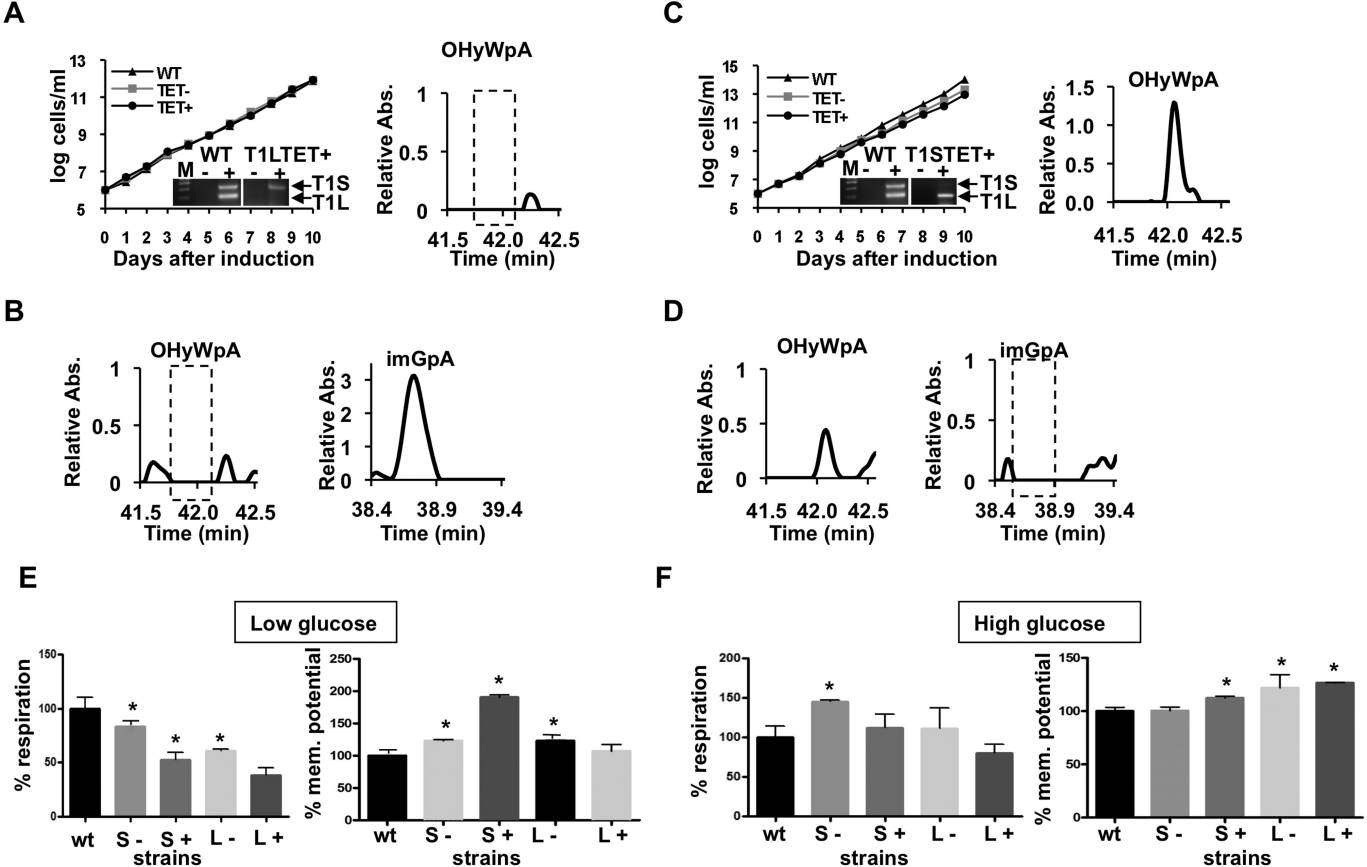
Two different wyosine derivatives co-exist in *T. brucei* mitochondria. (**A**) Growth curve of RNAi induced and uninduced TYW1L cells. The inset image shows RT-PCR analysis of TbTYW1L and TbTYW1S in WT and RNAi cells. TYW1L and TWY1S RT-PCR products are indicated by arrows (inset images). The right panel shows reduction of cytosolic hydroxywybutosine/adenosine dinucleotide monophosphate (OHyWpA) to undetectable levels by LC-UV-MS (dashed-line box). (**B**) mitochondrial RNA from the TbTYW1L RNAi-induced samples. The left panel shows loss of mitochondrial OHyWpA following RNAi of TYWL (left panel) but leads to slight increase of mitochondrial imGpA. (**C**) As in (**a**), showing growth curves for the RNAi-induced and uninduced TbTYW1S cell line. Down-regulation of TbTYW1S (right panel) has no effect on cytosolic OHyWpA. (**D**) Similar experiment with mitochondrial RNA fractions from the TbTYW1S RNAi cells. The left panel shows a 50% reduction in mitochondrial OHyWpA compared to the wild type, a complete disappearance of mitochondrial imGpA. In all panels ‘Relative abs’ refers to the ratio of the observed absorbance of a given peak from the RNAi lines, normalized to the same peak from the wild type, where identical amounts of tRNA were analyzed in all cases. The graphs shown are representative of at least 3 independent experiments. ‘log cells/ml’ refer to cumulative cell counts throughout the growth curve shown in days. ‘M’ in the inset images refers to a size marker used during electrophoresis. (**E**) shows respiration and membrane potential values determined when cells were grown in ‘Low-glucose’ media; these were compared with the same cell lines grown in normal media ‘High glucose’. Values shown are from at least three independent measurements. The measured mean values of red fluorescent intensity are represented as percentages of the WT sample, which was set to 100%. Standard deviations are indicated. A Student's *t*-test analysis determined that the results are significant, with *P* values of less than 0.05 (*). ‘wt’ refers to wild-type control, ‘S-’ and ‘L-’ refer to uninduced TbTYW1S and TbTYW1L cell lines respectively and ‘S+’ and ‘L+’ refer to cells in which RNAi was induced by tetracycline.

To further address the possibility of a wyosine biosynthetic pathway in mitochondria, we concentrated on the down regulation of TYW1L and TYW1S expression. We focused on these genes with the expectation that down-regulation of TYW1L would lead to disappearance of OHyW in the cytoplasm and mitochondrial fractions, in agreement with its synthesis in the cytosol followed by import of the hydroxywybutosine-containing tRNAs into the organelle; whereas down-regulation of TYWS would only affect formation of imG. This reasoning would be in accordance with the localization of these enzymes shown above. We generated individual transgenic RNAi cell lines. A portion of each gene (parts of the coding sequence) were placed in an RNAi plasmid vector under a tetracycline-inducible system, as previously described (25). Following RNAi induction by tetracycline, we performed growth curves to compare the un-induced and induced cell lines, followed by reverse transcription (RT)-PCR assays using primers specific for each gene (as described in ‘Materials and Methods’ section). By this approach, no products of each target gene were detected following RNAi induction, indicative of a successful down-regulation (Figure 5A and C; inset images). In these assays, RNA from wild type cells was used as a positive control, while a mock sample where reverse transcriptase was left out of the reaction served as a negative control for DNA contamination. To rule out secondary off-target effects between the two genes in question, in each reaction primers specific for the non-targeted TYW1 gene were included. In each case, RNAi induction led to the specific decrease in the levels of one transcript but not the other, while both transcripts were detected in the wild type sample (Figure 5).

To assess the effect of RNAi on wyosine and hydroxywybutosine biosynthesis, we then isolated subcellular fractions as before and compared the wyosine-derivative content of mitochondrial and cytosolic tRNA fractions, following RNAi induction (Figure 5). We observed a marked reduction in hydroxywybutosine in both fractions when TbTYW1L was down regulated (Figure 5A, right panel and 5 B, left panel). However, the content of wyosine in the mitochondrial fraction increased in comparison to wild type (Figure 5B, right panel). In turn, ablation of TbTYW1S led to a significant reduction in mitochondrial wyosine levels (Figure 5D, right panel) and only minor effects on mitochondrial hydroxywybutoisne (Figure 5D, left panel). These data indicate two separate functions for the two TYW1 paralogs: TbTYW1L is important for the presence of hydroxywybutosine and TbTYW1S is solely involved in wyosine formation. In turn, the observed reduction in mitochondrial hydroxywybutosine levels in the TbTYW1L RNAi-induced cells, together with the TbTYW1L localization experiments, is in line with its synthesis in the cytosol followed by import of the hydroxywybutosine-containing tRNAs into the organelle.

Down-regulation of expression of either gene does not cause a major growth defect (Figure 5A and C). This result is not surprising since modest growth phenotypes are observed with yW mutants in other organisms. However, upon ablation of either TbTYW1S or TbTYW1L, cells showed a growth phenotype in low-glucose media (Supplementary Figure S4D and S4E). This observation is significant because under low glucose cells are forced to grow primarily by oxidative phosphorylation. Therefore, TbTYW1S and TbTYW1L are important for mitochondrial function. This is also supported by an observed decrease in respiration and an increase in mitochondrial membrane potential upon RNAi induction in either cell line. These defects are more pronounced in the TbTYW1S-depleted cells (Figure 5E and F). In these experiments a reduction of growth in the uninduced samples was also observed. Although the leakiness of the RNAi approach is well documented in *T. brucei* (34), it is not clear why low glucose exacerbates leakiness in this particular system. Nonetheless, Measurement of specific mRNA down-regulation by quantitative PCR demonstrated that, at least, for TbTYW1S the difference in mRNA levels between induced (TET+) and uninduced (TET−) is significant, although the same may not be true for TbTYW1L (Supplementary Figure S4F and G). Although we do not presently understand the reasons for the increased membrane potential, coincidentally, it is analogous to what is observed upon TbTRM5 down-regulation (35). TbTRM5 is not only important to provide the m^1^G_37_ precursor for mitochondrial wyosine, but also for additional tRNAs.

### A novel eukaryotic pathway for wybutosine biosynthesis in *T. brucei* mitochondria

In light of these results, we propose a model for wyosine biosynthesis in *T. brucei* and perhaps in all kinetoplastids. Regardless of the intracellular compartment, the pathway starts with a tRNA substrate containing m^1^G_37_, which is produced by TbTRM5 (35). At this point, this substrate has two possible fates; a portion of it is retained in the cytosol and further modified by TbTYW1L, TbTYW2, TbTYW3A and TbTYW4/5, yielding OHyW_37_ as the end product. Some of this modified tRNA is then imported into the mitochondrion, explaining the presence of this modification in both compartments. This is consistent with the absence of TbTYW4/5 in mitochondria, which is absolutely required for the last step of OHyW formation, but not required for imG formation. Alternatively, either an m^1^G_37_-containing or the unmodified tRNA^Phe^ is imported into the mitochondrion and then modified by TbTYW1S; the former can directly partake in wyosine formation, the latter can be methylated to m^1^G_37_ once in the mitochondrion. This last proposal is in agreement with our previous description of a pathway for m^1^G_37_ methylation in the *T. brucei* mitochondrion (35).

The finding of a wyosine-modified dinucleotide containing adenosine (i.e. imGpA) suggests its presence in tRNA^Phe^, the only known substrate for wyosine derivatives in all organisms. In *T. brucei* of all the possible G_37_ containing tRNAs, tRNA^Phe^ is one of 3 that have a neighboring A_38_. However, this does not directly prove its existence in tRNA^Phe^ and it is possible that tRNAs other than tRNA^Phe^ may contain the modification. Unfortunately, it is not currently feasible to isolate sufficient amounts of tRNA^Phe^ from *T. brucei* mitochondria to perform direct sequencing by mass spectrometry and test this proposal. Instead, however, we resorted to purified tRNA^Phe^ from mitochondrial and/or cytoplasmic fractions isolated from the closely related trypanosomatid *Leishmania tarentolae*, using oligonucleotide-affinity hybridization, as described elsewhere (36). The *L. tarentolae* genome encodes the same set of duplicated genes yielding LtTYW1L and LtTYW1S (accession number: LtaP5.0770 and LtaP15.1500 respectively) with the same predicted intracellular localization (one cytoplasmic and the other mitochondrial). The *L. tarentolae* purified tRNA^Phe^ was analyzed by LC-MS/MS to confirm the presence of the modifications at position 37. The resulting spectra are consistent with the presence of OHyW_37_ in tRNA^Phe^ from the cytosolic tRNA fraction (Supplementary Figure S5A) and imG_37_ or OHyW_37_ in the mitochondrial fraction (Supplementary Figure S5B). We also found yW_37_-containing tRNA^Phe^. In our scheme above, this may represent an intermediate in the cytosolic pathway given that TbTYW4 is not a mitochondrial enzyme (Supplementary Figure S5B).

**Figure 6.**
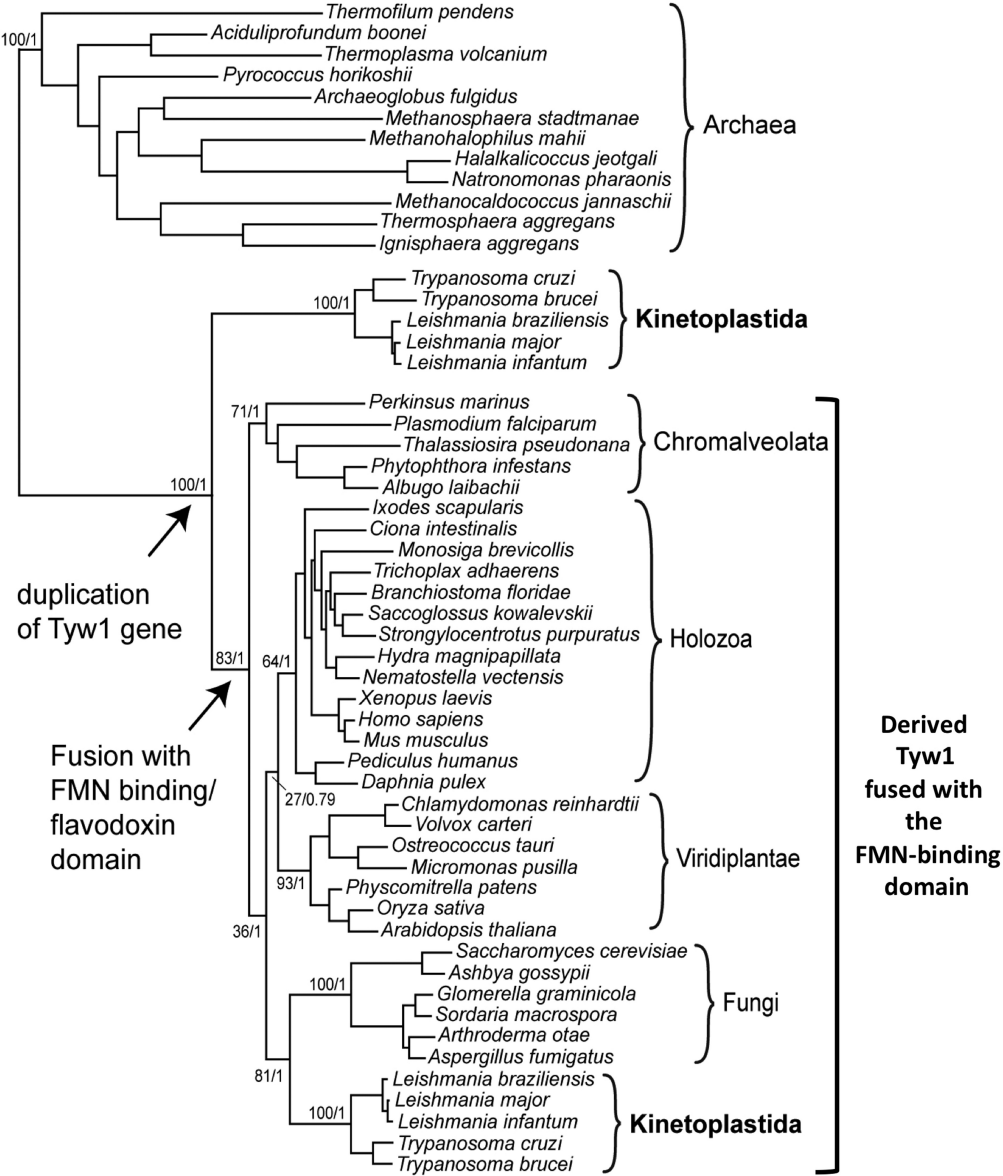
TbTY1S and TbTyW1L are the result of a gene duplication event. Maximum likelihood (ML) phylogenetic tree of TAW1/TYW1 protein sequences excluding the flavodoxin domain. Numbers at nodes are ML bootstrap/Bayesian posterior probability values and are shown only for relevant branches representing major taxonomic groups and the relationships among them. The two major events in the evolution of the eukaryotic TYW1 are highlighted by arrows. FMN refers to the Flavin Mononucleotide-binding domain.

Unlike TAW1 of Archaea, most of the known eukaryotic TYW1 proteins possess N-terminal flavodoxin domains. However, as shown here, parasitic trypanosomatids of the group Kinetoplastida encode additional variants that lack this domain. To explore how the eukaryotic pathway may have evolved, we performed phylogenetic analysis of TAW1/TYW1 proteins. For the eukaryotic TYW the flavodoxin domain was excluded from the alignment to prevent biasing the tree towards relationships arising from the conservation of this domain, which as discussed is not found in all of the proteins in question (Figure 6). This analysis was performed with several different algorithms; all yielding similar tree topologies. The analysis strongly supports the idea of a gene duplication event in the eukaryotic lineage that involved acquisition of an FMN domain that then gave rise to extant FMN-containing TYW1 enzymes.

## DISCUSSION

Presented here is the first description of wyosine derivatives in organellar tRNA. Co-existence of two diverging pathways in *T. brucei* (and presumably related species) opens a window into the possible evolutionary origin of wyosine/wybutosine biosynthesis. Notably, the mitochondrial TbTYW1S resembles the archaeal enzyme, while TbTYW1L is in line with those cytosolic pathways described in other eukaryotes. Various scenarios could explain the occurrence of such a wyosine synthesizing activity in organelles. One possibility is that a gene for a wyosine biosynthetic enzyme existed in the bacterial ancestors of mitochondria and this gene, like many others, was eventually transferred to the nuclear genome where it acquired a mitochondria-targeting signal. However, there is no evidence for the existence of a wybutosine pathway in any sequenced bacterial genome or for that matter in bacterial tRNAs. Thus, we deem a bacterial origin unlikely. A more likely scenario is that the FMN-containing proteins are, according to our phylogenetic tree, derived from the non-FMN-containing TYW1S (bootstrap values of 100% and a Bayesian probability of 1). A third possibility is that given the similarity between TYW1S and TAW1 (the archaeal counterpart), the eukaryotic enzyme was horizontally acquired from the Archaea. However, TbTYW1S forms its own clade within the eukaryotic lineage (Figure 6). In addition, none of the eukaryotic enzymes clusters with the Archaea, ruling out acquisition by horizontal gene transfer. Lastly, it is still possible that the FMN-lacking enzyme dates back to last common ancestor of Archaea and Eukarya (Last Eukaryotic Common Ancestor, LECA), we cannot support or disprove this possibility with our phylogenetic analysis partly due the degree of divergence of these enzymes.

Whether or not the *T. brucei* TYW1S is derived from Archaea or predated the eukaryal/archaeal split is not currently clear, taken together, we suggest that the extant cytosolic pathway is the result of a more recent acquisition of the FMN-binding module by TbTYW1L in *T. brucei* and TYW1 in other eukaryotes. By extension, the newly discovered mitochondrial pathway described here, in analogy to Archaea, should require a transacting reductase to provide the essential FMN-domain function. Along these lines, as mentioned before in *C. utilis* a cytosolic pathway for imG already exists, providing an example of an organism where the gene duplication did not occur, although one cannot formally rule out the possibility of a secondary loss of the FMN-containing enzyme in *C. utilis*, and for that matter, in *T. brucei* mitochondria. The *cis*-acting FMN-domain may have provided a catalytic advantage for the cyclization reaction, obviating the need for the second gene, which eventually led to its disappearance. Fusion of the enzymes and the proteins that serve as their electron donors or acceptors (e.g. flavodoxin, cytochrome, etc.) is not without precedent, for example, some fatty acid desaturases use soluble cytochrome b5 as electron donor while in others cytochrome b5 is fused to the enzyme (37).

Wyosine biosynthesis shows great diversity between different organisms, most prominently in Archaea; yet the presence of multiple pathways occurring in a single species as shown here for trypanosomes is unprecedented. By far, the most chemically intricate frameshift-preventing modification at position 37 involves the nucleoside wyosine (imG) and its derivatives, including wybutosine (yW) and hydroxywybutosine (OHyW) (5) in tRNA^Phe^. Wyosine derivatives are exclusively found in Archaea and Eukarya. This unique evolutionary conservation may be related to the observed propensity of ribosomes to shift reading frames during translation in response to ‘slippery’ sequences, most prominently those rich in uridines and consequently also rich in Phe codons. Evidence suggests that wybutosine promotes stable codon-anticodon pairing by base stacking interactions with anticodon loop nucleotides (5). Therefore, the potential frameshifting problem caused by U-rich slippery sequences may be partly solved by the unique presence of wyosine and its derivatives in mitochondria. This we have demonstrated with mitochondrial tRNA^Phe^ of *Leishmania tarentolae*, while in the specific case of *T. brucei*, because of the inherent difficulties in purifying sufficient material for mass spectrometry analysis, establishing the presence of imG in tRNA^Phe^ will remain an open question.

These observations lead to several important questions of why trypanosomatids maintain the ancestral pathway and dedicate it to their mitochondria. This may be the result of yet another twist of trypanosomatid mitochondrial evolution, going back to the appearance of the robust, highly complex and prevalent U insertion/deletion RNA editing mechanism. Mitochondria-encoded mRNAs in trypanosomatids and related flagellates are transcribed as ‘scrambled’ sequences that undergo, sometimes extensive, insertions and deletions of uridines (38). The obvious end result is the generation of edited translatable messages. A less appreciated facet of editing is that in generating uridine-rich sequences, it may pose the mitochondrion with the danger of potential defects in reading-frame maintenance, as shown for cytosolic systems. Hence, the wyosine pathway might have been recruited to the mitochondria to avoid translational errors.

Why then the imported wybutosine-containing tRNA is not sufficient for mitochondrial translation? In this respect the very robust mitochondrial tRNA import pathway, in combination with a level of promiscuity by the import machinery, allows for the transport of both fully matured as well as undermodified tRNAs. In a previous report, we presented evidence showing that undermodified tRNAs are imported into the mitochondrion and then ‘recycled’ by mitochondrial modification, making them available for translation (35). In this case, we suggest that recycling involves wyosine formation, elegantly avoiding potential translational defects but necessitating maintenance of at least a portion of the pathway. Unfortunately, the lack of tractable mitochondrial genetics or an *in vitro* translation system makes it currently impossible to test these ideas directly. Still, we examined the possible biological significance of hydroxywybutosine/wyosine in mitochondrion by analyzing the effect of ablation of either TbTYW1L or TbTYW1S. Although only minor effects on cell growth and mitochondrial function are observed under standard conditions, when the same cells were grown in low glucose medium, which demands mitochondrial function, either enzyme becomes important for growth.

More globally in most cases mutants that do not make yW, m^1^G_37_ alone is sufficient to ameliorate frameshifting. Why has m^1^G not universally replaced yW? The answer may rest in the idea of ‘frameshifting potential’, whereby, like in many viruses, frameshifting is used in a programmed manner, perhaps to increase coding diversity (39). The levels of either wyosine or derivatives may fluctuate with changes in environmental conditions leading to more or less frequent frameshifting as needed to create protein diversity and alter cell function. This may be especially true in trypanosome mitochondrion where ‘alternative’ editing has been invoked as a possible source of coding diversity (40), yet coding diversity may be also achieved with the newly discovered wyosine pathway presented in this work.

Beyond the argument for accuracy during protein synthesis, these observations offer an interesting tale about the evolution of translational systems and cells in general. It is clear that modifications such as wyosine/hydroxywybutosine were the invention of the Archaea and Eukarya to the exclusion of the bacterial lineage. Surprisingly, the bacterial translation system of the trypanosomatid mitochondrion has adopted this purely non-bacterial modification to perhaps ensure reading-frame maintenance in the organelle; highlighting the vagaries that cellular systems endure once endosymbiosis and natural selection ensues.

## SUPPLEMENTARY DATA

Supplementary Data are available at NAR Online.

SUPPLEMENTARY DATA
