# Supplementary material for: A common tRNA modification at an unusual location: the discovery of wyosine biosynthesis in mitochondria
Source: Nucleic Acids Res. 2015 Apr 6;43(8):4262–73. doi: 10.1093/nar/gkv286 (PMC4417183; doi:10.1093/nar/gkv286)
Supplement: SUPPLEMENTARY DATA [file supp_43_8_4262__index.html]

A common tRNA modification at an unusual location: the discovery of wyosine biosynthesis in mitochondria — A common tRNA modification at an unusual location: the discovery of wyosine biosynthesis in mitochondria — SUPPLEMENTARY DATA 

# A common tRNA modification at an unusual location: the discovery of wyosine biosynthesis in mitochondria

## SUPPLEMENTARY DATA

**Files in this Data Supplement:**

- SUPPLEMENTARY DATA
