# Supplementary material for: A common tRNA modification at an unusual location: the discovery of wyosine biosynthesis in mitochondria
Source: Nucleic Acids Res. 2015 Apr 6;43(8):4262–73. doi: 10.1093/nar/gkv286 (PMC4417183; doi:10.1093/nar/gkv286)
Supplement: SUPPLEMENTARY DATA [file supp_gkv286_nar-00587-r-2015-File008.pdf]

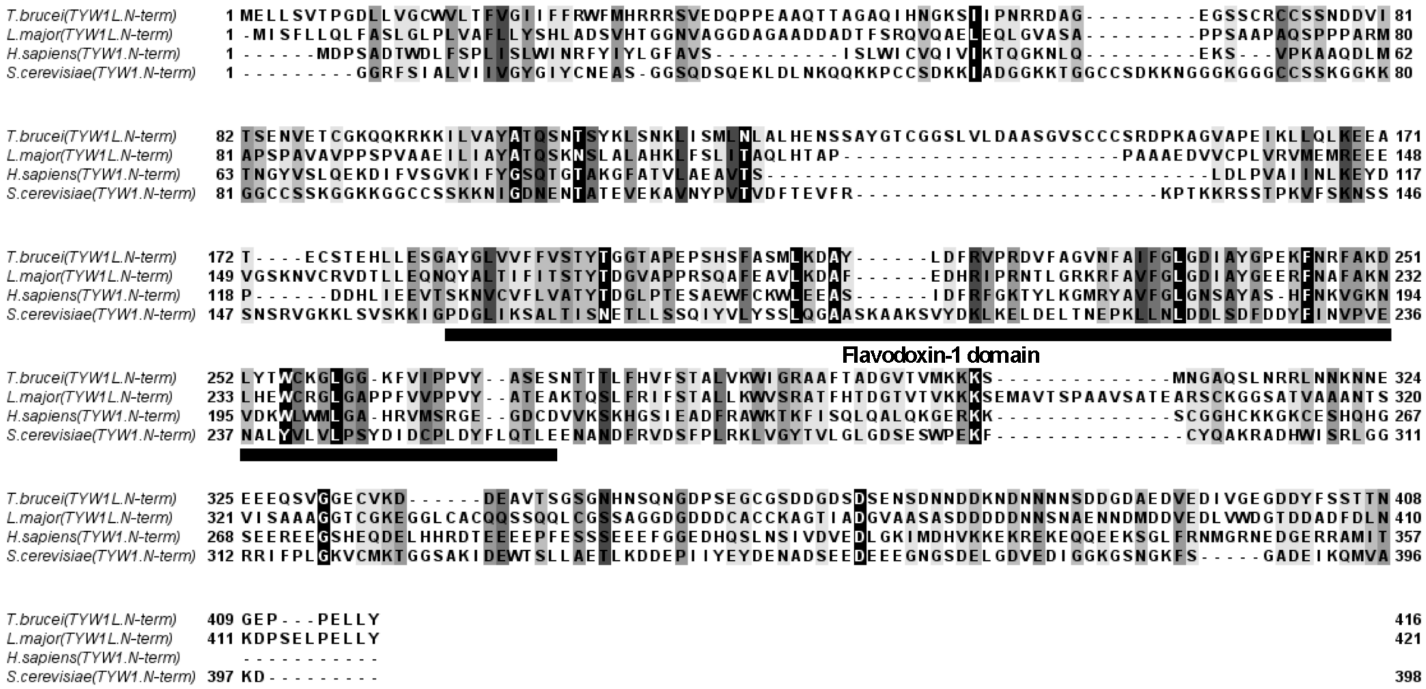

Supplementary Figure 1A

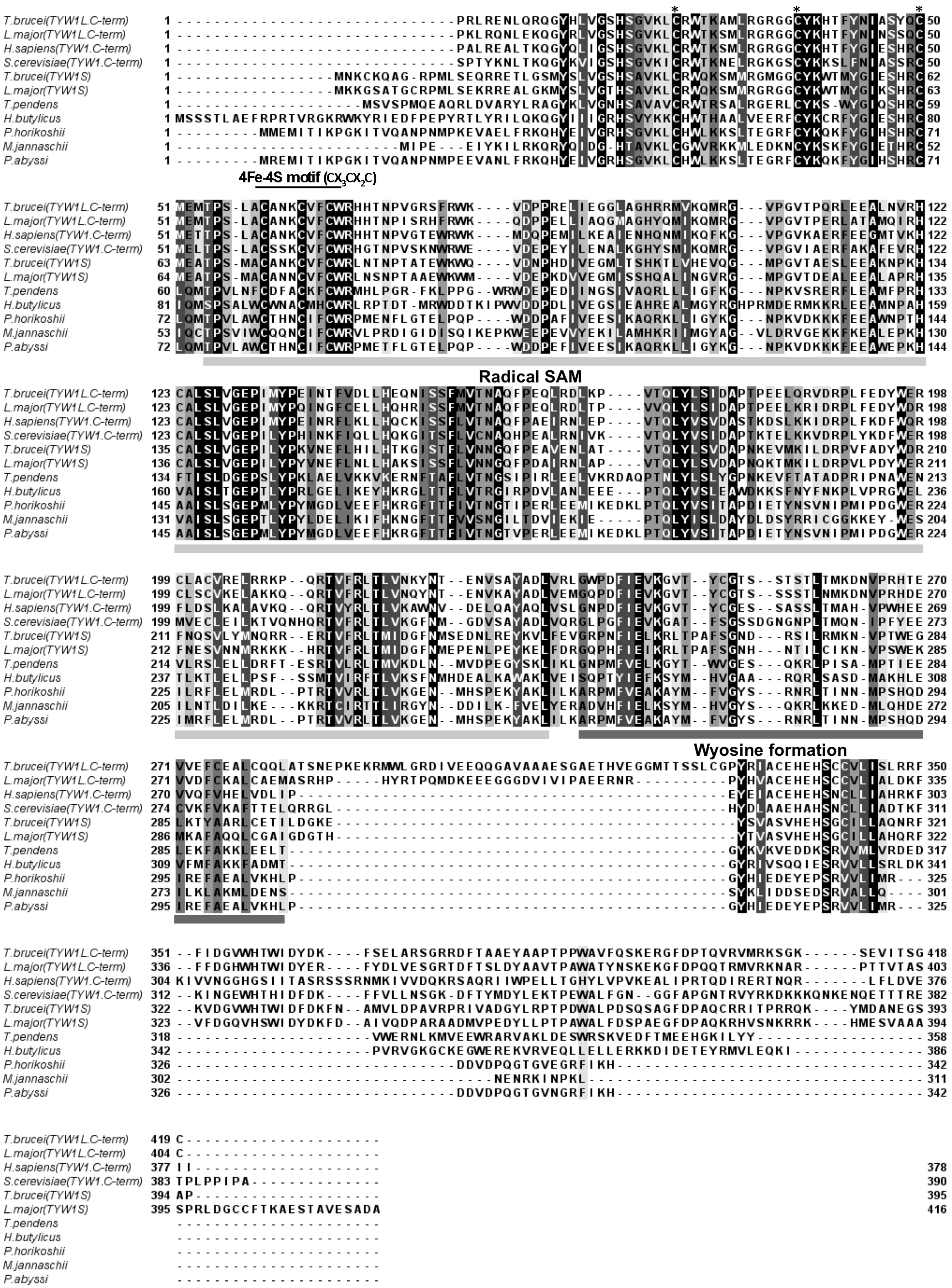

Supplementary Figure 1B

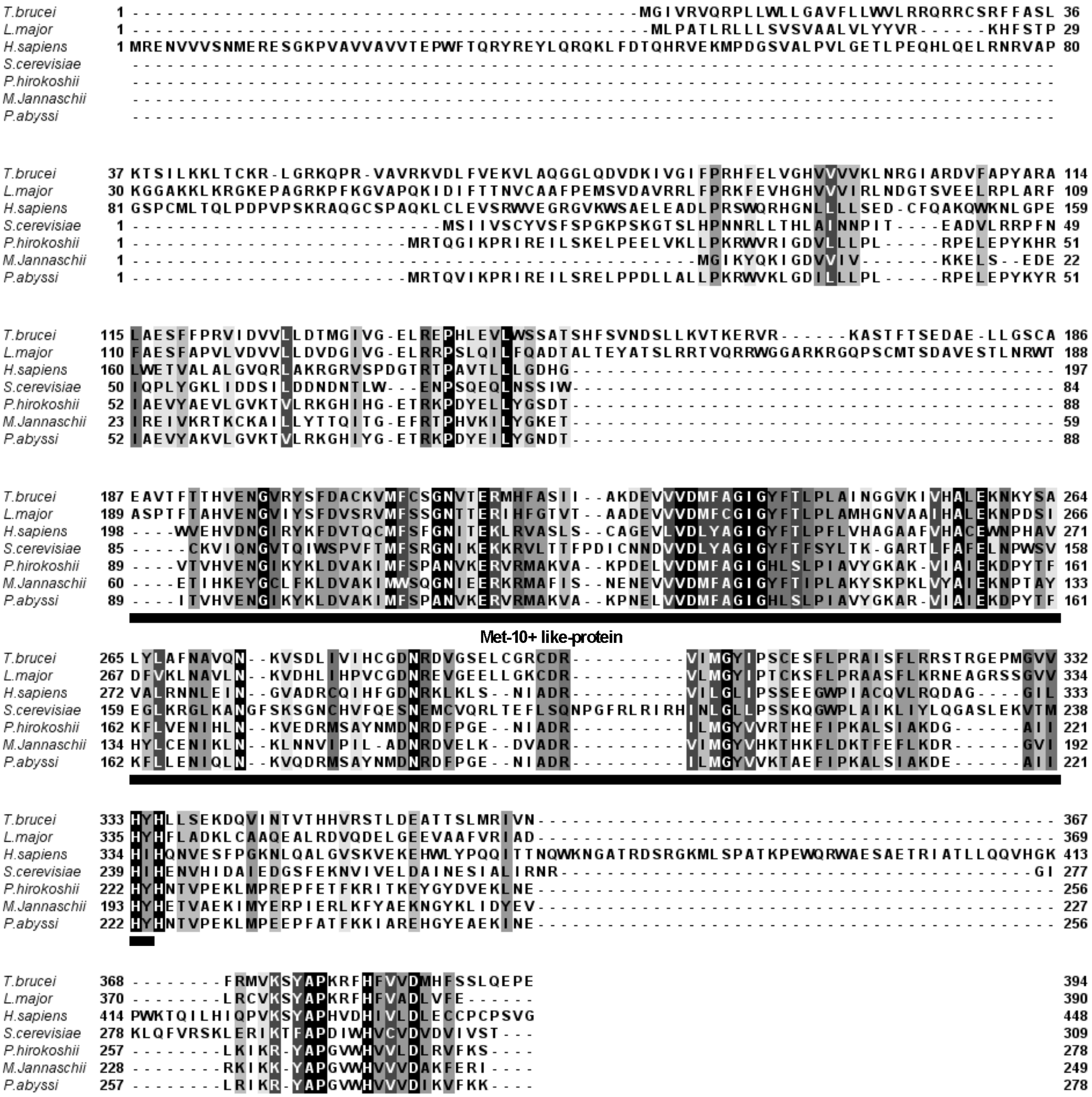

Supplementary Figure 1C

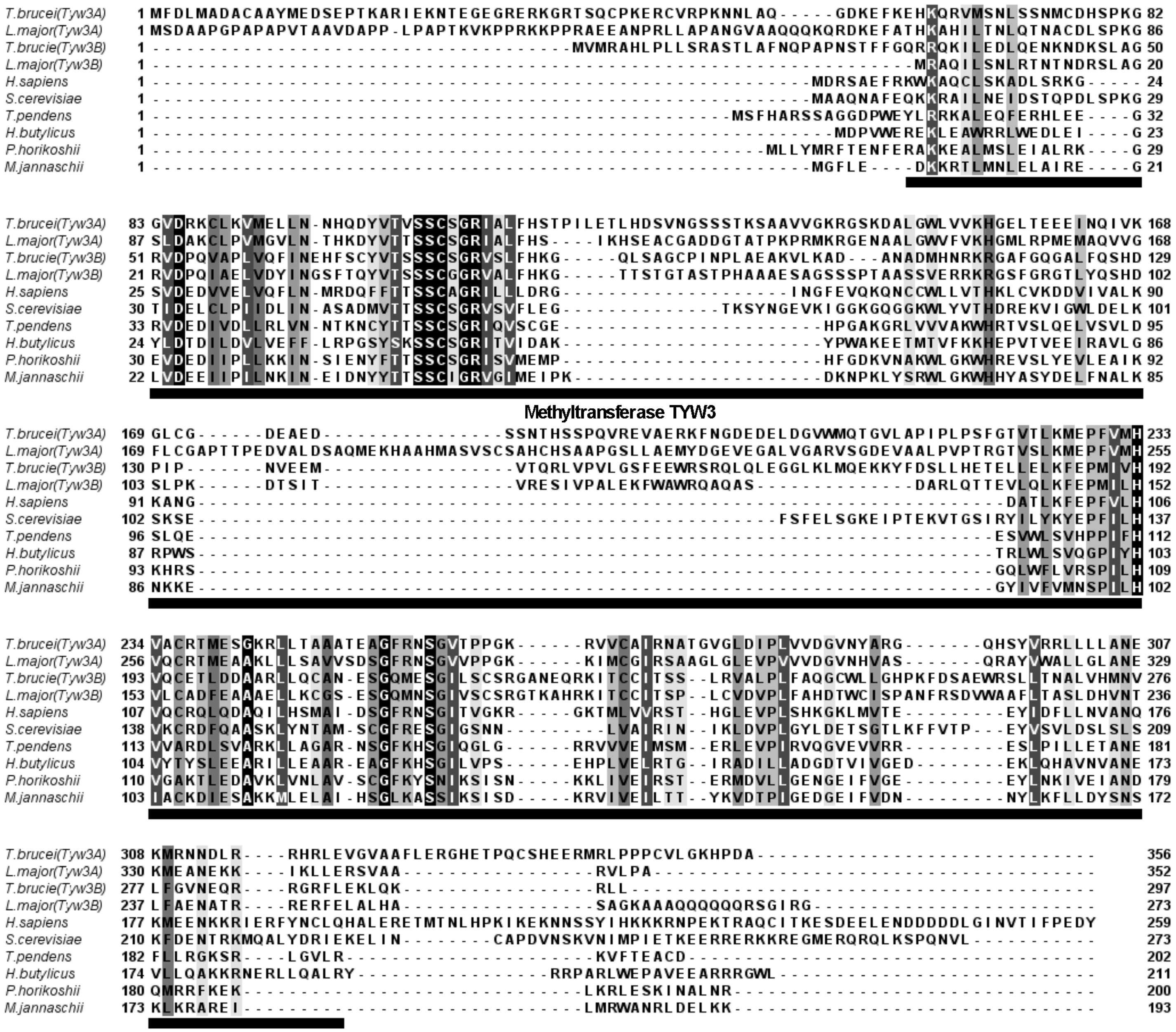

Supplementary Figure 1D

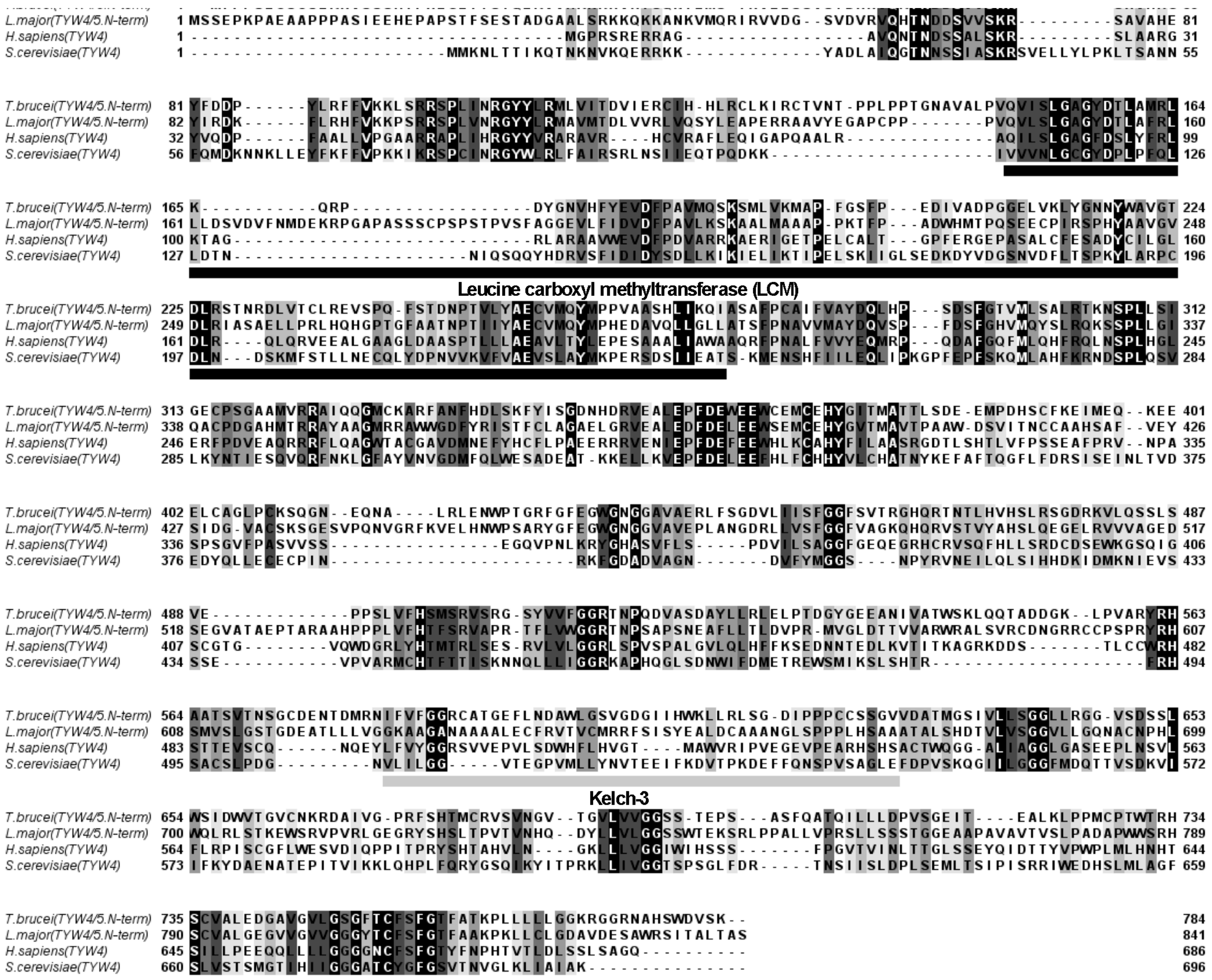

Supplementary Figure 1E

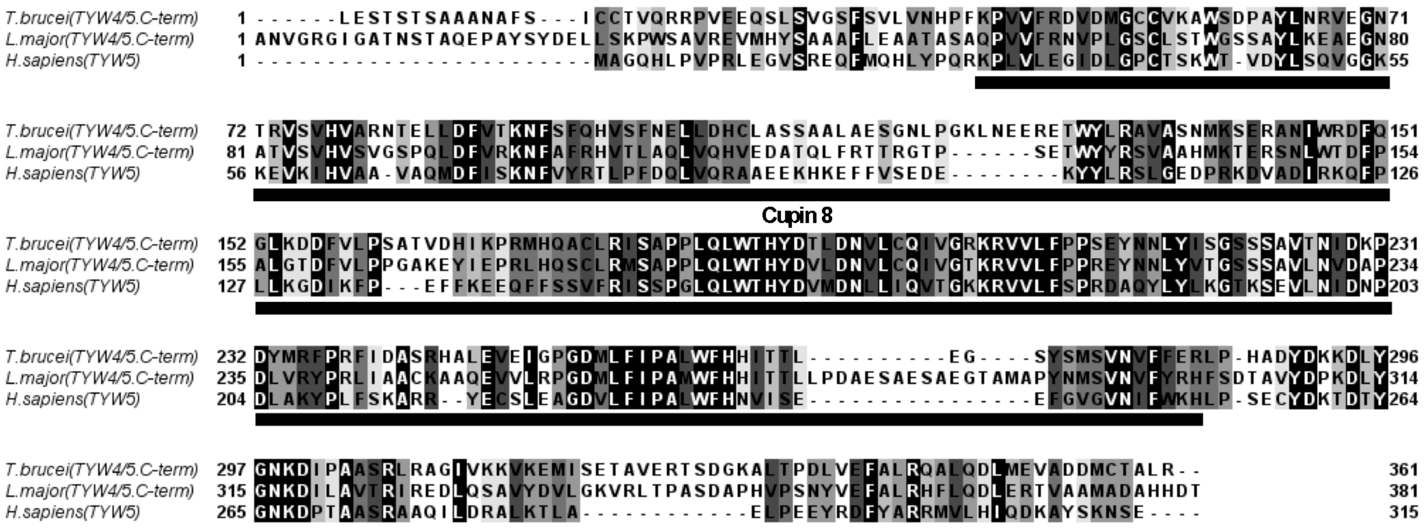

Supplementary Figure 1F

A

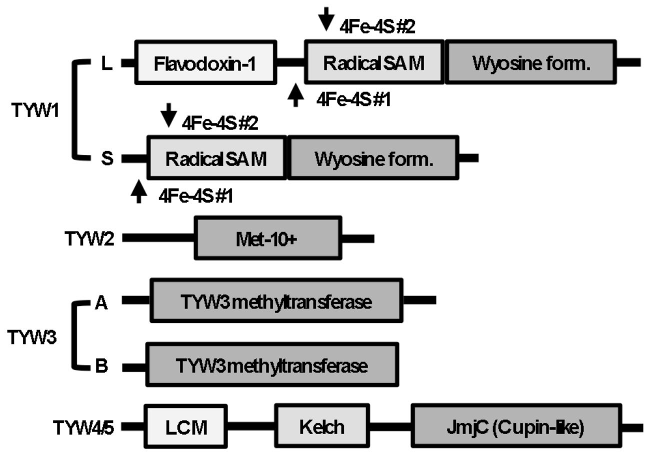

B

	TargetP Prediction						Experimentally Determined
	mTP	SP	Other	Loc	RC	Tplen	
Tyw1L	0.061	0.863	0.079	S	2	35	**cytosolic**
Tyw1S	0.664	0.055	0.307	M	4	63	**mitochondrial**
Tyw2	0.509	0.76	0.005	S	4	29	**cytosolic and mitochondrial**
Tyw3A	0.048	0.091	0.947	-	1	-	**cytosolic**
Tyw3B	0.755	0.084	0.113	M	2	24	**mitochondrial**
Tyw4	0.621	0.029	0.357	M	4	13	**cytosolic**

Supplementary Figure 2

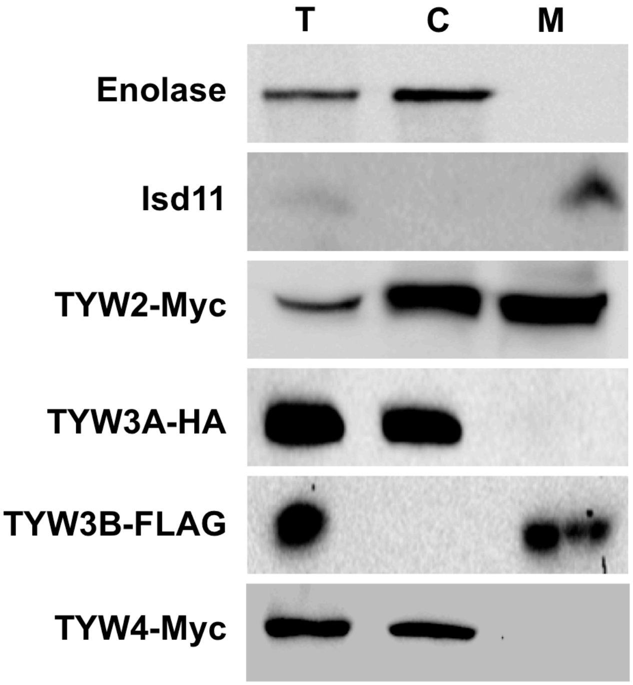

Supplementary Figure 3

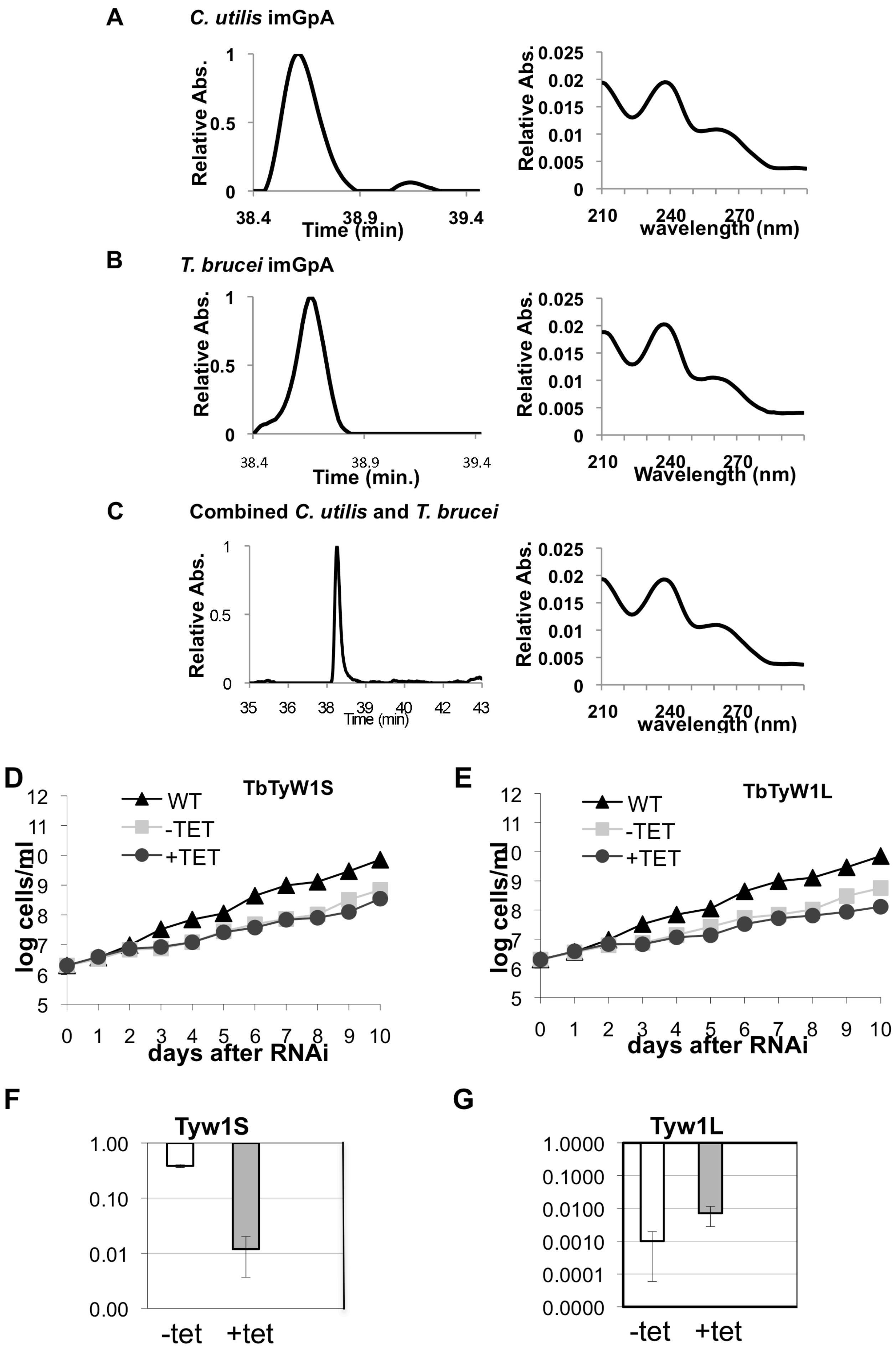

Supplementary Figure 4

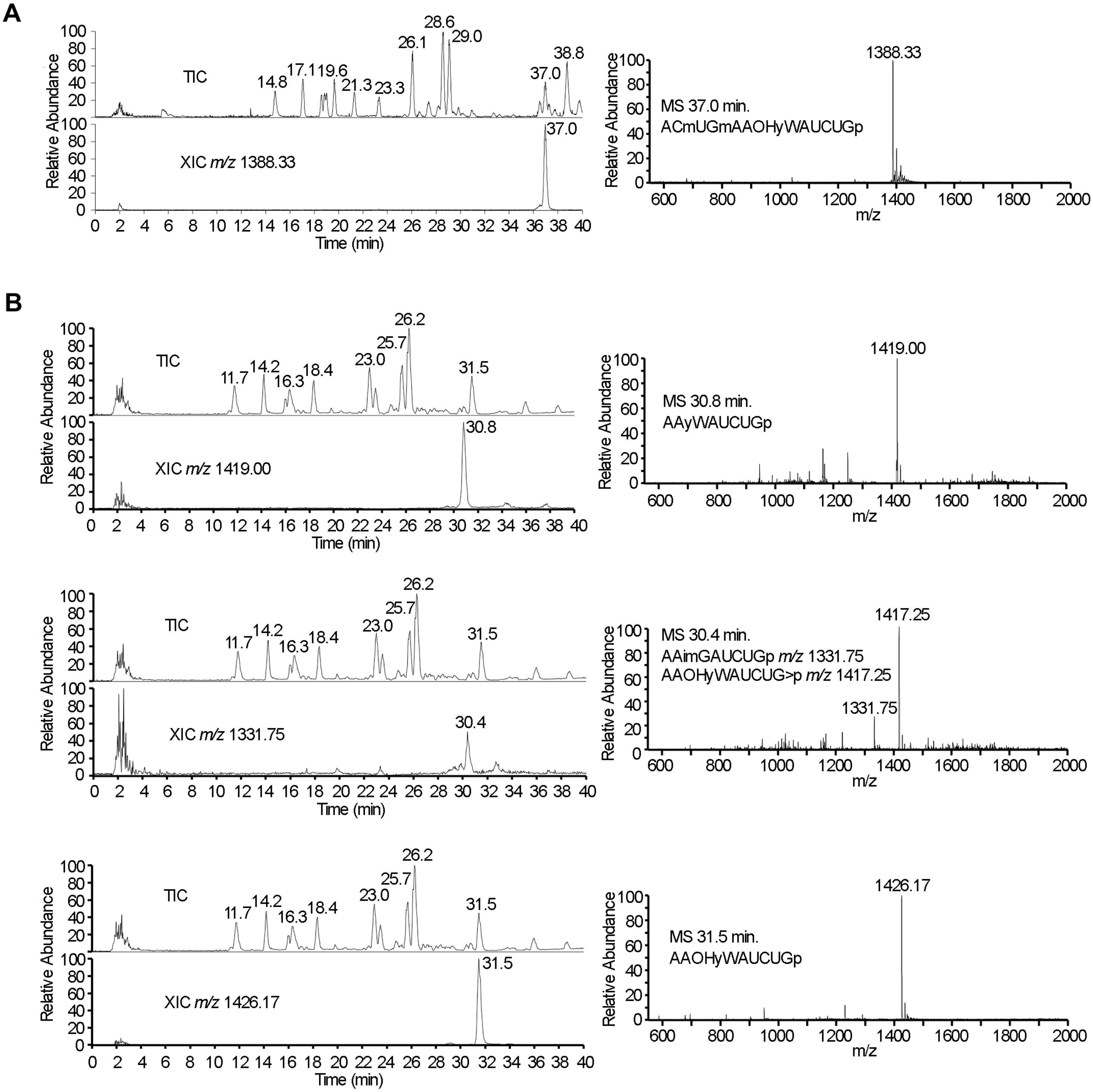

Supplementary Figure 5

Supplementary Figures

Supplementary Figure 1 | Sequence Alignment of Kinetoplastid TYW protein sequences with archaeal and other eukaryotic homologs. (**a**) The N-terminus of eukaryotic TYW1 (including kinetoplastid TYW1L) contains a flavodoxin-1 domain, which binds a flavin mononucleotide (FMN) that reduces the N-terminal iron-sulfur cluster. Archaeal TYW1 homologs do not contain a flavodoxin-1 domain and the hypothetical trans-acting factor for archaeal TYW1 iron-sulfur cluster reduction remains unknown. (**b**) Alignment of the C-terminus of eukaryotic TYW1 (including kinetoplastid TYW1L), full length TYW1 from archaea and kinetoplastid TYW1S. Asterisks indicate conserved cysteine residues in the two iron-sulfur clusters (4Fe-4S). One iron sulfur cluster preceding the radical SAM domain is coordinated by the $CX_{12}CX_{12}C$ motif; while the adjacent iron-sulfur cluster is coordinated by a CX_3CX_2C motif. Using PFAM, protein domains for 'radical SAM' and 'wyosine formation' were identified in each sequence, while the flavodoxin-1 domain was found only in eukaryotic TYW1 homologs. (**c**) Alignment of eukaryotic and archaeal TYW2. (D) Alignment of eukaryotic and archaeal TYW3, including the two kinetoplastid TYW3 paralogs. (E) Alignment of the N terminus of the kinetoplastid TYW4/5 and full length eukaryotic TYW4. TYW4 homologs have not been identified in Archaea. (F) Alignment of the C terminus of kinetoplastid TYW4/5 and full-length *Homo sapien* TYW5.

Supplementary Figure 2 | Graphic representation of the *T. brucei* wybutosine-synthesizing homologs. (**a**) The two TYW1 paralogs differ with respect to one protein domain - flavin mononucleotide (FMN) binding domain (or flavodoxin-1) is present at the N terminus of TYW1L and absent in TYW1S. TYW4 and TYW5 are fused in trypanosomes, while in mammals they exist as separate proteins. (**b**) Subcellular localization prediction by TargetP. mTP: Amino acid score based on known mitochondrial targeting sequences. SP: Strength of any present secretory pathway sequences. Other: Strength of localization prediction toa location other than the mitochondrion or secretory pathway. Loc: Final localization prediction based on the scores of 'mTP', 'SP', and 'Other'. S: secretory pathway. M: mitochondrion. '-' : other. RC (reliability class): Confidence of localization prediction based on the difference between the highest and second highest localization scores. 1 is the highest and 5 is the lowest. TPlen: number of amino acids residues between the N-terminus and a predicted presequence cleavage site. '-' indicates that a cleavage site was not predicted.

Supplementary Figure 3 | Subcellular localization of TbTYW2, TbTYW3A, TbTYW3B, and TbTYW4/5. Western blot analysis of epitope-tagged constructs from total, cytosolic, and mitochondrial protein fractions. Enolase is used as a cytosolic marker and as a means to determine mitochondrial fraction purity. Isd11 serves as both a mitochondrial marker and a control for cytosolic fraction purity. TbTYW2-Myc localizes to both the cytosolic and mitochondrial fractions. TbTYW3A-HA is localized to the cytosol, while TbTYW3B-FLAG is strictly found in the mitochondrion. TbTYW4/5-Myc is strictly cytosolic.

Supplementary Figure 4 | Positive identification of wyosine, and absence of isowyosine, in the trypanosome mitochondrion. (**a**) imGpA (wyosine-phosphate adenosine) from *Candida utilis* elutes at 38.7 minutes (left panel) and has the absorbance profile seen in the right panel. (**b**) Wyosine from *T. brucei* elutes at 38.7 minutes and has a matching absorbance profile to the *C. utilis* wyosine absorbance profile. (**c**) Combination of *C. utilis* imGpA fraction and *T. brucei* suspected imGpA fraction co-elute. The combined C. utilis and *T. brucei* sample is shifted slightly to the left, likely due to the nearly complete lack of other nucleosides that would influence (retard) the imGpA dinucleoside as it migrates through the reverse-phased HPLC column. (**d**) and (**e**) Effects of TbTyW1S and TbTyW1L RNAi on the growth of the procyclic stage of *T. brucei* in the low glucose media compared to normal media as indicated. Growth curves of wild type (WT; triangles), non-induced (TET-; squares) and induced (TET+; circles) knockdown cell line for TbTyW1S and TbTyW1L. The y axis is labeled by a log scale and represents the products of the measured cell densities and total dilutions. Cell densities were measured using the Beckman Z2 cell counter.

Supplementary Figure 5 | Sequence-determining ions observed in product ion mass spectra from collision-induced dissociation of *L. tarentolae* tRNA^{Phe} RNase T1-derived anticodon oligonucleotides. (**a**) Cytoplasmic tRNA^{Phe} (**b**) mitochondrial tRNA^{Phe}. The conventional tRNA numbering has been used throughout, yielding the sequence AA(OHyW$_{37}$/yW$_{37}$/imG$_{37}$)AUCUG as indicated. All c- and y-type fragment ions and 4 of 5 possible w-type ions were detected and used to confirm the sequence. Only oligonucleotides covering the anticodon loop including position 37 are shown.

Supplementary Information methods

Western Blot

The protein coding regions with different epitope tags, were generated for the other *T. brucei* wybutosine-synthesizing genes: TYW2-Myc, TYW3A-HA, TYW3B-FLAG, and TYW4-Myc. These constructs were individually transformed into procyclic *T. brucei* 29-13 cells and clonal cell lines were established as described before. Expression was induced by the addition of tetracycline for 24 hr and either harvested and total cell extracts prepared (as described elsewhere) to use for Western blot analysis.

For each construct, one liter of induced cells were harvested at 1 x 10^7 cells/ml. 50 ml of this culture was sonicated to produce the total cell lysate. The rest of the culture was used to prepare the cytosolic and mitochondrial fractions as described elsewhere (36). Total, cytosolic, and mitochondrial protein fractions (10 mg/lane) were separated on 10% SDS–polyacrylamide gel, blotted, and subjected to Western blot analysis with the following monoclonal mouse primary antibodies: V5 and FLAG (Sigma-Aldrich), Myc (Abcam), HA (Sigma), and His (Santa Cruz). Secondary anti-mouse IgG antibodies coupled to horseradish peroxidase (GE Healthcare) were use for visualization using the Clarity™ Western ECL system (BioRad) and following the manufacturer's instructions. Rabbit polyclonal antibodies specific for *T.* brucei Isd11 (28)

and enolase (kindly provided by P.A.M. Michels) were used as controls for mitochondrial and cytosolic fraction purity, respectively.
